# Fibrome non ossifiant à localisation rare à propos d'un cas

**DOI:** 10.11604/pamj.2016.23.163.8945

**Published:** 2016-04-06

**Authors:** Adil El Alaoui, Ilyas Rabhi

**Affiliations:** 1 Service de Chirurgie Orthopédique (A), Centre Hospitalier Universitaire Hassan II de Fès, Maroc

**Keywords:** Fibrome, bénigne, humérus, Fibroma, benign, humerus

## Image en médecine

Il s'agit d'une patiente âgée de 26 ans, sans antécédentsparticuliers, qui présente depuis 2 mois des douleurs localisées au niveau de la partie proximale de l'humérus droit irradiantesvers l’épaule sans notion de fièvre ou d'altération de son état général. Chez qui l'examen Clinique trouve une tuméfaction en regard de la face externe du bras gauche douloureuse à la palpation sans signes inflammatoires en regard. Une radiographie et scanner de l'humérus ont objectivé une image lacunaire ostéolytique au niveau du tiers proximal avec effraction de la corticale interne (A,B). Le bilan d'extension n'a pas montré d'autres localisations. La patiente a bénéficié dans un premier temps d'une biopsie de la tumeur revenant en faveur d'une tumeur bénigne type fibrome non ossifiant, puis dans un deuxième temps d'une exérèse de la tumeur associée à une synthèse par clou verrouillé et comblements du vide par du ciment (C).

ése par clou verrouillé et comblement du vide par du ciment

**Figure 1:**
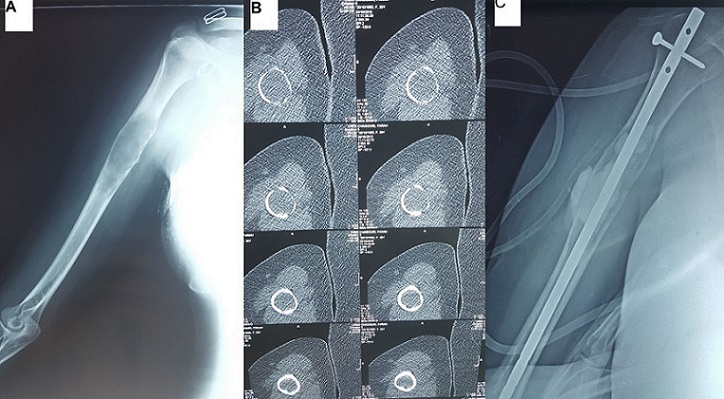
A) radiographie de l'humérus droit montrant une image lacunaire ostéolytique au niveau du tiers proximal; B) à scanner de l'humérus droit montant une image ostéolytique avec éffraction de la corticale; C) radiographie de controle de l'humérus droit après biopsie éxerese de la tumeur associée à une ostéosynth

